# Biochemical and structural characterization of quizalofop-resistant wheat acetyl-CoA carboxylase

**DOI:** 10.1038/s41598-021-04280-x

**Published:** 2022-01-13

**Authors:** Raven Bough, Franck E. Dayan

**Affiliations:** Department of Agricultural Biology, Colorado State University, Fort Collins, CO 80523 USA

**Keywords:** Enzyme mechanisms, Biochemistry, Plant sciences, Plant biotechnology, Plant physiology

## Abstract

A novel nucleotide mutation in *ACC1* resulting in an alanine to valine amino acid substitution in acetyl-CoA carboxylase (ACCase) at position 2004 of the *Alopecurus myosuroides* reference sequence (A2004V) imparts quizalofop resistance in wheat. Genotypes endowed with the homozygous mutation in one or two *ACC1* homoeologs are seven- and 68-fold more resistant to quizalofop than a wildtype winter wheat in greenhouse experiments, respectively. In vitro ACCase activities in soluble protein extracts from these varieties are 3.8- and 39.4-fold more resistant to quizalofop with the homozygous mutation in either one or two genomes, relative to the wildtype. The A2004V mutation does not alter the specific activity of wheat ACCase, suggesting that this resistance trait does not affect the catalytic functions of ACCase. Modeling of wildtype and quizalofop-resistant wheat ACCase demonstrates that the A2004V amino acid substitution causes a reduction in the volume of the binding pocket that hinders quizalofop’s interaction with ACCase. Docking studies confirm that the mutation reduces the binding affinity of quizalofop. Interestingly, the models suggest that the A2004V mutation does not affect haloxyfop binding. Follow up in vivo and in vitro experiments reveal that the mutation, in fact, imparts negative cross-resistance to haloxyfop, with quizalofop-resistant varieties exhibiting higher sensitivity to haloxyfop than the wildtype winter wheat line.

## Introduction

Acetyl-CoA carboxylase (ACCase or EC 6.4.1.2) catalyzes the first committed and rate-limiting step of fatty acid biosynthesis, converting acetyl-CoA to malonyl-CoA^1^. The three primary functional components of ACCase are biotin carboxylase, biotin carboxyl carrier protein, and carboxyl transferase (see Figure 1 in Takano et al., 2021)^2^. Mechanistically, biotin carboxylase utilizes ATP to facilitate the carboxyl group transfer from bicarbonate to the biotin cofactor. The biotin carboxyl carrier protein then moves the newly acquired carboxyl group to the carboxyl transferase domain, where acetyl-CoA is carboxylated to form malonyl-CoA. Most plants have two forms of ACCase, a eukaryotic multifunctional homomeric form located in the cytosol and a prokaryote-like multi-subunit form localized to plastids^1^. Grasses differ from most plants by having only the eukaryotic form of the enzyme in both the cytosol and plastids^3,4^. Group 1 herbicides specifically target the eukaryotic form of ACCase by binding to the carboxyl transferase domain and inhibiting the second catalytic step^5^, whereas these herbicides have little activity on the multi-subunit chloroplastic form. Consequently, group 1 herbicides are excellent graminicides^6^.

The recently commercialized CoAXium® winter wheat production system consists of a specialized quizalofop formulation under the tradename Aggressor® for use on wheat varieties with the AXigen® quizalofop resistance trait coupled with a stewardship program aiming to delay resistance in weeds^7^. Quizalofop, applied as the proherbicide form quizalofop-p-ethyl, controls grasses postemergence. Following cuticle penetration, the proherbicide is bioactivated to the active form quizalofop acid *in planta*. Plants transport the active quizalofop to meristematic tissue, which contains a large concentration of plastid-bound ACCase. Plastid-bound wheat ACCase encoded by the gene *ACC1* occurs as a homodimer with three functional domains per monomer^8,9^. Although the binding of quizalofop to ACCase has not been studied previously, work on other aryloxyphenoxy-propionate herbicides demonstrates that these are reversible, noncompetitive inhibitors of grass ACCase^10,11^. All ACCase inhibitors (aryloxyphenoxy-propionates, cyclohexandiones and phenylpyrazolines) interact with the same binding domain on the carboxyl transferase portion of the multifunctional form of ACCase^12,13^.

The AXigen® trait is a mutant quizalofop-resistant wheat ACCase. Researchers discovered the trait via ethyl methanesulfonate (EMS) mutagenesis and subsequent screening for resistance to quizalofop^14^. The nucleotide substitution in *ACC1* imparting quizalofop resistance in CoAXium® wheat corresponds to position 2004 in the ACCase amino acid sequence from the *Alopecurus myosuroides* reference sequence^14^. This mutation causes an amino acid substitution of an alanine for a slightly larger valine residue in ACCase (A2004V). Initially, the researchers identified three accessions with heterozygous resistance-conferring mutations for each of three *ACC1* gene homoeologs in hexaploid wheat (6x = 42) on chromosome 2 and later developed a line with a homozygous mutation for each homoeolog. Not all homoeologs contribute equally to quizalofop resistance, with the A to V mutation on the D homoeolog providing the greatest level of resistance, followed by the same mutation in the A and B homoeologs. The Wheat Breeding and Genetics Program at Colorado State University introgressed the mutations from the A and D *ACC1* homoeologs into an elite genetic background and developed the initial CoAXium® varieties featuring the mutation in both homoeologs.

Our objectives were to determine if the amino acid substitution affects the specific activity of wheat ACCase and to describe how the substitution imparts resistance at biochemical and structural levels. We further quantified fold-differences in whole-plant and enzymatic resistance between wheat with wildtype ACCase and wheat with one or two mutant ACCase homoeologs.

We first determined whole-plant quizalofop resistance in wheat with one or two mutant ACCase homoeologs by comparison to wildtype, susceptible wheat and correlated these experiments to the effect of quizalofop in in vitro assays of wheat ACCase. We then modeled the changes in the ACCase protein structure and its interaction with the herbicide using homology modelling, molecular dynamic simulations, and docking.

## Results

### Herbicide dose effect on plant growth

Three wheat genotypes were grown in the greenhouse to assess the contribution of a homozygous *ACC1* mutation in either one or two genomes leading to whole-plant quizalofop resistance relative to the wildtype line, and to examine cross-resistance to haloxyfop. In general, whole-plant resistance to either quizalofop or haloxyfop varies by the number of mutant *ACC1* homoeologs present in a wheat genotype. The residual standard error of the 2-parameter log-logistic regression model with Box-Cox transformation of quizalofop doses is 11.5 with 97 degrees of freedom. For the haloxyfop doses, the residual standard error of the 4-parameter Brain-Cousens model with Box-Cox transformation is 2.14 with 92 degrees of freedom.

**Figure 1:**
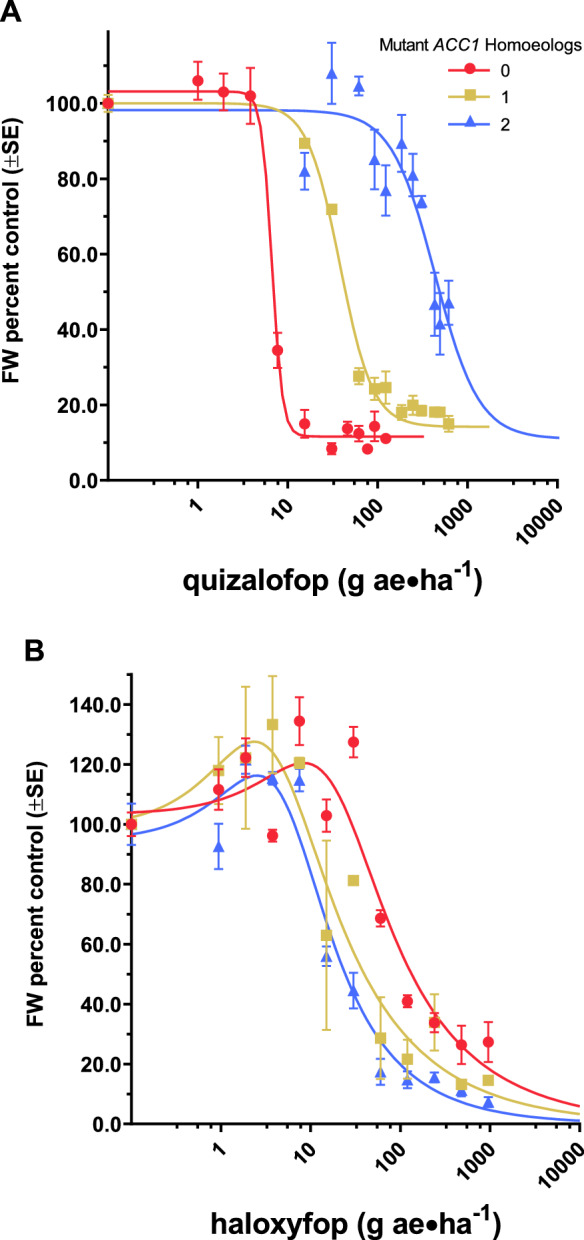
Models^a^ of quizalofop (**A**) and haloxyfop (**B**) herbicide dose effects on whole-plant growth by genotype^b^. ^a^Leaf fresh weight values (g) normalized to percent untreated controls and transformed with Box-Cox transformation per genotype for each herbicide. Quizalofop dose effect modeled with a 2-parameter log-logistic function (**A**) and haloxyfop dose effect modeled with a 4-parameter Brain-Cousens function (**B**). ^b^Genotype refers to the number of homozygous mutations in *ACC1* that result in a A2004V amino acid substitution, with up to three possible on group 2 chromosomes in hexaploid wheat (2n = 6x). These mutations result in quizalofop-resistant wheat ACCase.

The estimated quizalofop doses that reduce fresh biomass growth normalized as a percent of untreated controls by 50% (GR_50_) range from 7.13 to 485 g ae ha^−1^, while haloxyfop GR_50_ values range from 26.5 to 125 g ae ha^−1^ (Table 1 and Fig. 1). Based on GR_50_ values, genotypes with the homozygous mutation in either one or two genomes are 7.0 and 68.0 times more resistant to quizalofop than the wildtype, susceptible genotype, respectively. The quizalofop-resistant genotypes are more sensitive to haloxyfop than the wildtype. Lines with the mutation in either one or two of the genomes are about three-fold and five-fold more sensitive to haloxyfop, respectively, relative to the susceptible genotype (Table 1). According to Student’s t-tests, these genotypes are significantly different from the susceptible genotype for both herbicides.

**Table 1:** Relative herbicide dose effects on whole-plant growth by genotype^a^ for 50% growth reduction (GR_50_)^b^.

Herbicide ae	Mutant *ACC1* Homoeologs	GR_50_ (g ae ha^−1^)	R:S^c^	R:S *p*-value^d^
Quizalofop	0	7.13 (1.0)	–	–
	1	50.1(5.8)	7.0 (1.31)	< 0.0001
	2	485 (43.6)	68.0 (11.6)	< 0.0001
haloxyfop	0	125 (28.7)	–	–
	1	40.4 (13.4)	0.32 (0.130)	< 0.0001
	2	26.5 (6.1)	0.21 (0.07)	< 0.0001

^a^Genotype refers to the number of homozygous mutations in *ACC1* that result in a A2004V amino acid substitution, with up to three possible on group 2 chromosomes in hexaploid wheat (2n = 6x).

^b^Leaf fresh weight values (g) normalized to percent untreated controls and transformed with Box-Cox transformation per genotype for each herbicide. Quizalofop dose effect modeled with a 2-parameter log-logistic function and haloxyfop dose effect modeled with a 4-parameter Brain-Cousens function to estimate GR_50_ values.

^c^Resistance factor, calculated by dividing the GR_50_ of a quizalofop-resistant genotype by the susceptible genotype GR_50_.

^d^Student’s t-test *p*-value (α = 0.05) of resistance factor ratios.

### Enzyme specific activity assay

**Figure 2:**
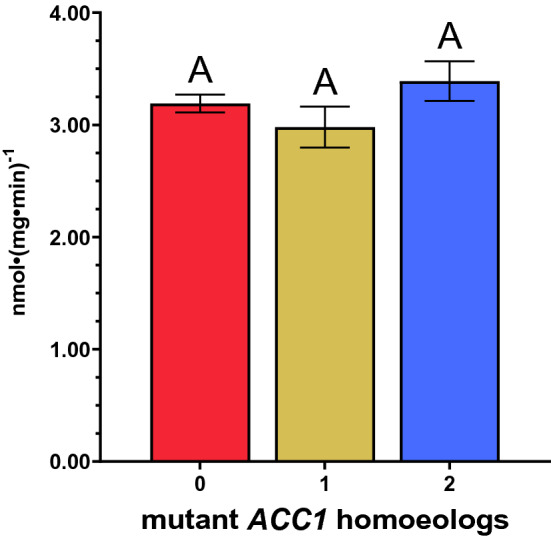
ACCase specific activity by genotype^a,b^. ^a^Genotype refers to the number of homozygous mutations in *ACC1* that result in a A2004V amino acid substitution, with up to three possible on group 2 chromosomes in hexaploid wheat (2n = 6x). These mutations result in quizalofop-resistant wheat ACCase. ^b^Genotype specific activities labeled with the same capital letter are not significantly different according to Student’s t-tests (α = 0.05).

Crude enzyme preparations containing ACCase activity, the target enzyme of quizalofop and other group 1 herbicides, were extracted from wheat to compare specific activity and to determine enzyme-level resistance of the same three genotypes used in the whole-plant resistance studies. Enzyme extract specific activity range from 2.66 to 3.91 nmol mg protein^−1^ (Fig. 2). Values are not significantly different between genotypes (F_2,6_ = 0.278).

### Herbicide dose effect on ACCase activity

Enzyme-level resistance to either quizalofop or haloxyfop varies by the number of mutant *ACC1* homoeologs present in a wheat genotype. The residual standard error of the 2-parameter log-logistic regression model with Box-Cox transformation of quizalofop doses is 0.435 with 48 degrees of freedom. A similar model of haloxyfop doses has a residual error of 0.940 with 55 degrees of freedom.

**Figure 3:**
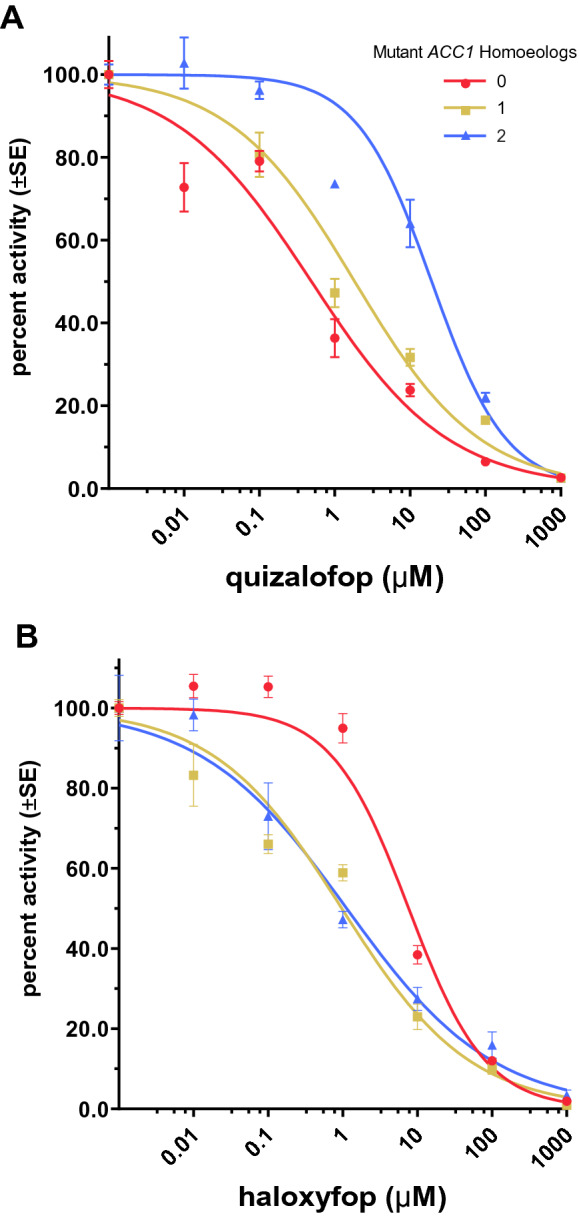
Models^a^ of quizalofop (**A**) and haloxyfop (**B**) herbicide dose effects on ACCase activity by genotype^b^. ^a^Genotype refers to the number of homozygous mutations in *ACC1* that result in a A2004V amino acid substitution, with up to three possible on group 2 chromosomes in hexaploid wheat (2n = 6x). These mutations result in quizalofop-resistant wheat ACCase. ^b^Activity values (DPM) normalized to percent untreated controls and transformed with Box-Cox transformation per genotype for each herbicide. The dose effect of each herbicide is modeled with a 2-parameter log-logistic function.

The estimated quizalofop doses that inhibit ACCase activity normalized as a percent of untreated controls to 50% (I_50_) range 0.486 to 19.3 µM, while haloxyfop I_50_ values range from 0.968 to 7.63 µM (Table 2 and Fig. 3). ACCase extracts from invididuals with the resistance mutation in one or two genomes are 3.8 and 39.4 times more resistant to quizalofop than the wildtype, susceptible genotype, respectively, based upon estimated I_50_ values. Conversely, ACCase extracts from the same quizalofop-resistant genotypes are less resistant to haloxyfop than the susceptible genotype. These genotypes are between seven- and eight-fold more sensitive to haloxyfop than the wildtype (Table 2 and Fig. 3).

**Table 2:** Relative herbicide dose effects on ACCase activity by genotype^a^ for 50% inhibition (I_50_)^b^.

Herbicide ae	Mutant *ACC1* Homoeologs	I_50_ (µM)	R:S^c^	R:S *p*-value^d^
Quizalofop	0	0.49 (0.13)	–	–
	1	1.84 (0.52)	3.8 (1.5)	0.0634
	2	19.30 (4.01)	39.4 (13.3)	0.00571
Haloxyfop	0	7.63 (1.08)	–	–
	1	0.97 (0.21)	0.13 (0.003)	< 0.0001
	2	1.12 (0.25)	0.15 (0.004)	< 0.0001

^a^Genotype refers to the number of homozygous mutations in *ACC1* that result in a A2004V amino acid substitution, with up to three possible on group 2 chromosomes in hexaploid wheat (2n = 6x). These mutations result in quizalofop-resistant wheat ACCase.

^b^Activity values (DPM) normalized to percent untreated controls and transformed with Box-Cox transformation per genotype for each herbicide. The dose effect of each herbicide is modeled with a 2-parameter log-logistic function to estimate I_50_ values.

^c^Resistance factor, calculated by dividing the I_50_ of a quizalofop-resistant genotype by the susceptible genotype I_50_.

^d^Student’s t-test *p*-value (α = 0.05) of resistance factor ratios.

While the resistance factor for lines with the AXigen trait in two genomes is statistically significant for quizalofop in Student’s t-test, the resistance factors for lines with a single resistant homoeolog is not statistically significant although the *p*-value is close to the significance threshold. Resistance to haloxyfop in these genotypes is significantly different from the susceptible genotype.

The resistant factors at the enzyme level are approximately half of those measured on whole plant dose–response curves (Tables 1 and 2). The higher level of resistance *in planta*, relative to the in vitro activity, is common because plants have additional mechanisms to cope with the presence of herbicides, such as reduced foliar absorption and metabolic degradation of xenobiotics^15^.

Nonetheless, our results suggest that quizalofop resistance conferred by the AXigen® trait is additive at both the whole-plant and enzyme-level. Estimated GR_50_ values in the whole-plant dose response trials for the susceptible and single-mutant genotype were within confidence intervals provided by Ostlie et al. in 2015^14^, however, our resistance factor estimate for the single-mutant genotype based on these values is nearly double.

Wheat lines with the quizalofop-resistance trait are more sensitive to haloxyfop relative to the susceptible genotype in both whole-plant and enzyme-level dose–response trials. These results suggest that while the ACCase conformational change induced by the A2004V amino acid substitution increases quizalofop-resistance, the change causes negative cross-resistance to haloxyfop (e.g., increases susceptibility to haloxyfop). The negative cross-resistance to haloxyfop is not concerning for the CoAXium® Wheat Production System because the system is only available in the United States, where haloxyfop is not labeled for use in wheat.

### Homology modeling of wheat carboxyl transferase domain and docking of quizalofop and haloxyfop

Both quizalofop and haloxyfop are group 1 herbicides that target multifunctional eukaryotic forms of ACCase. However, our biological studies in the greenhouse and biochemical characterization of ACCase reveal that the alanine to valine mutation imparts tolerance to quizalofop both *in planta* and in vivo, while causing negative cross-resistance to haloxyfop.

**Figure 4:**
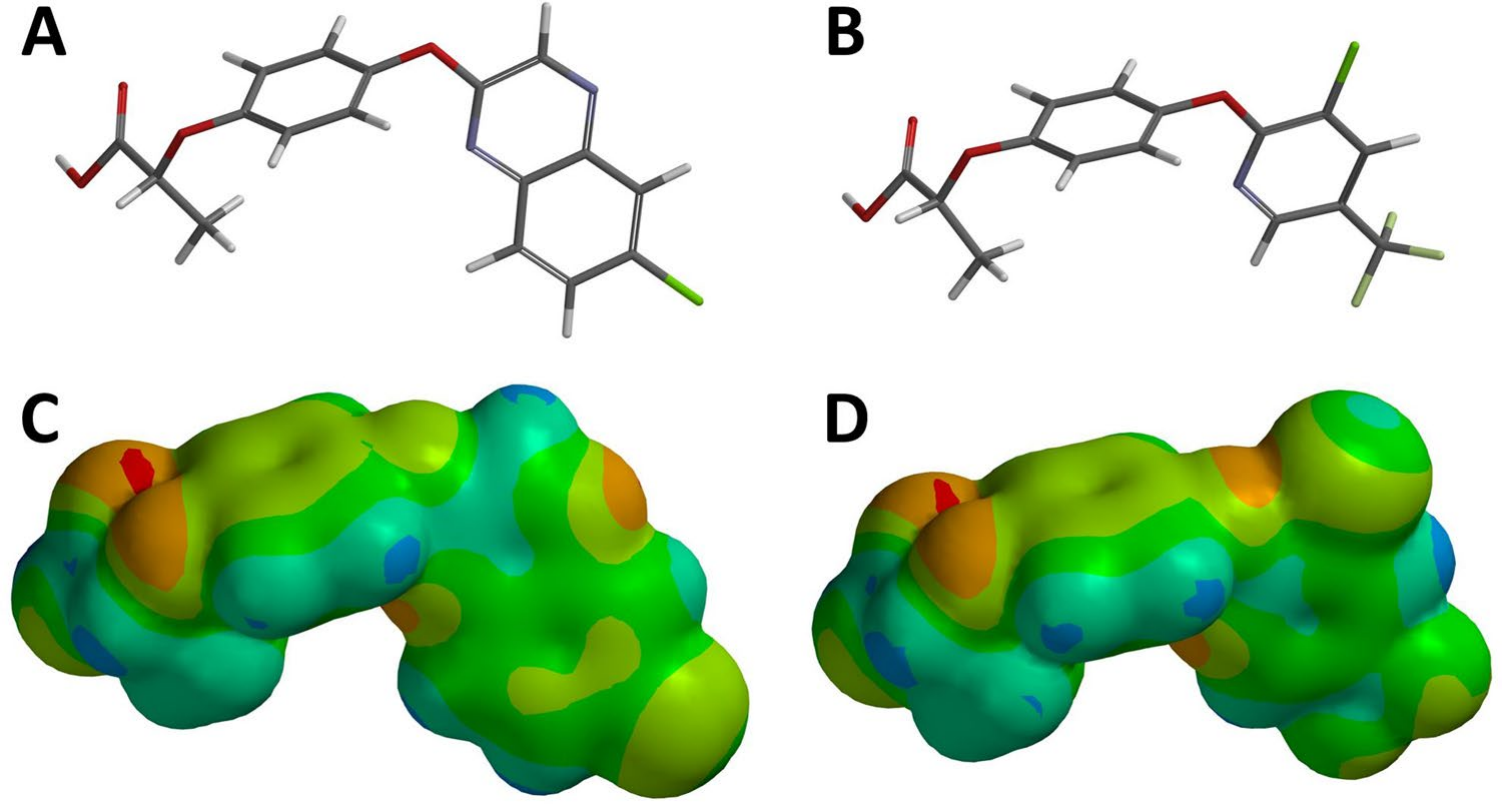
Structures of (**A**) quizalofop and (**B**) haloxyfop showing the carbons in gray, hydrogen in white, oxygen in red, nitrogen in blue, chlorine in green and fluorine in cyan on the left side. Electrostatic potential maps of (**C**) quizalofop and (**D**) haloxyfop. Colors on the volume represent range from electronegative (red) to electropositive (blue) partial charges. Images were generated using Spartan’20 version 1.1.2 (Wavefunction, Inc., Irvine, CA 92612) (https://www.wavefun.com/).

Although quizalofop and haloxyfop are structurally related (Fig. 4A,B), quizalofop is a slightly larger molecule due to its heterobicyclic 6-chloro-quinoxalin-2-yl rings relative to the monocyclic 3-chloro-5-(trifluoromethyl)pyridin-2-yl ring of haloxyfop, with CPK volumes of 303.97 and 317.09 A^3^, respectively (Table 3). Quizalofop has a smaller molecular weight than haloxyfop despite a larger volume. Both herbicides are lipophilic, although quizalofop is more so according to a greater partition coefficient (log*P*). Quizalofop has a slightly larger dipole moment than haloxyfop, indicating it is more polar. The herbicide molecules have a similar electrostatic potential (Fig. 4C,D).

**Table 3:** Physicochemical properties of quizalofop and haloxyfop.

Parameters	Quizalofop	Haloxyfop
Mw	344.75	361.70
log*P*	1.89	0.65
CPK volume A^3^	317.09	303.97
Energy (1000 kj mol^−1^)	− 4006.2	− 4445.5
Dipole moment	3.51	3.08

**Figure 5:**
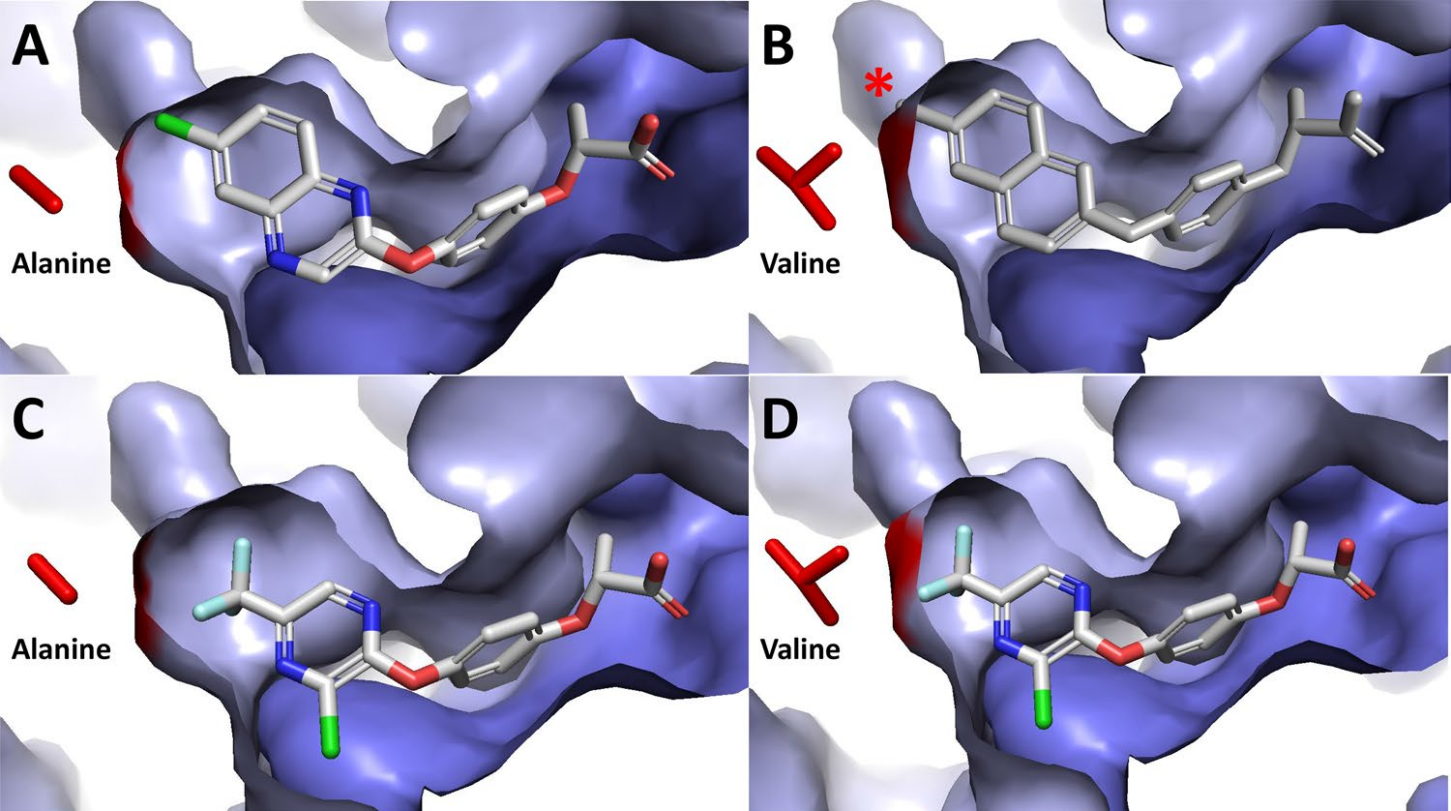
Conformational change caused by the alanine to valine amino acid substitution at position 2004 affects the binding of quizalofop to the carboxyl transferase domain of ACCase. Docking of quizalofop and haloxyfop in the wild-type wheat carboxyl transferase domain are shown in panels (**A**,**C**) with the methyl group of the alanine side chain shown in red. Changes to the binding cavity caused by the valine substitution (isopropyl group in red) are shown in panels (**B**,**D)**. The small reduction in the volume of the binding domain introduces strong steric hinderance (as denoted by asterisk) that prevents quizalofop from binding (panel **C**). The structure of quizalofop is shown in white solely illustrate the steric hindrance because the herbicide no longer binds in this domain. On the other hand, the valine mutation does not interfere with haloxyfop binding (panel **D**). Images were generated using PyMOL version 2.3.3 (Schrödinger, New York, NY 10036) (https://www.schrodinger.com/products/pymol).

The calculated binding energy of quizalofop increases from − 6.07 to − 5.70 kcal mol^−1^, whereas the calculated binding energy of haloxyfop decreases slightly from − 5.074 to − 5.211 kcal mol^−1^ (Table 4). The number of correct docking poses and root-mean-square deviation of atomic poses for all docking combinations are within acceptable limits. Clearly, both quizalofop and haloxyfop bind to the catalytic domain of the wildtype carboxyl transferase (Fig. 5A,C). The A2004V amino acid substitution is located at the bottom left of the herbicide binding domain, where the methyl side chain of alanine occupies a smaller volume than that of the isopropyl side chain of valine. The asterisk in Fig. 5B highlights the strong steric hindrance between the volume occupied by the valine side chain and the 6-chloro-quinoxalin-2-yl group of quizalofop. The same A2004V amino acid substitution does not prevent binding of haloxyfop (Fig. 5D).

**Table 4:** Docking parameters of quizalofop and haloxyfop on wheat ACCase.

	Quizalofop	Haloxyfop
	Wildtype wheat ACCase	
Correct docking poses	92/110	82/110
Calculated binding energy^a^	− 6.070 ± 0.095	− 5.074 ± 0.206
RMSD^b^	0.273 ± 0.021	0.245 ± 0.033

	Quizalofop-resistant wheat ACCase	
Correct docking pose	95/110	104/110
Calculated binding energy	− 5.700 ± 0.230	− 5.211 ± 0.221
RMSD	0.434 ± 0.034	0.242 ± 0.035

^a^Calculated binding energy in kcal mol^−1^.

^b^RMSD relative to initial coordinate of quizalofop or haloxyfop based on the crystal structure of 1UYS.

## Discussion

Group 1 herbicides are reversible, noncompetitive inhibitors of the multifunctional form of ACCase^10,11^. Since grass plants only possess this form, group 1 herbicides are excellent graminicides^16,17^. Whole-plant quizalofop dose greenhouse experiments confirm resistance in quizalofop-resistant wheat genotypes (Table 1). Dose–response curves shift right with an increasing number of *ACC1* resistance alleles (Fig. 1A), therefore having the mutation in two genomes is more resistant than if present in a single genome, and the level of quizalofop resistance imparted by the AXigen® trait appears to be additive. The level of resistance to quizalofop at the enzyme-level parallels the plant responses in the greenhouse study (Table 2 and Fig. 3A), establishing a strong linear relationship between the number of mutant resistant homoeologs with the phenotypic and enzymatic responses.

The lack of significant differences in specific activity between genotypes with susceptible *ACC1* and one or two resistant *ACC1* homoeologs suggests that the conformational change induced by the A2004V amino acid substitution does not affect ACCase activity. This supports field trials data reporting comparable CoAXium® wheat yields following quizalofop treatment to non-treated, quizalofop-susceptible wheat yields (data not shown).

Several structures of the multifunctional form of ACCase have been co-crystalized with inhibitors from the three main families of herbicides from this group (i.e., aryloxyphenoxy-propionates, cyclohexandiones and phenylpyrazolines). Structures reveal that these inhibitors hold a similar pose in the binding domain on the carboxyl transferase portion of the multifunctional form of ACCase^12,13^. The location of the A2004V mutation imparting resistance to quizalofop in wheat is within the same binding domain targeted by ACCase inhibitors^12,13^. While an amino acid substitution from alanine to valine does not affect the overall architecture of the carboxyl transferase domain, it imparts a local conformational change that reduces the volume of the binding pocket. This relatively small change impacts the binding of quizalofop because of its large heterobicyclic 6-chloroquinoxaline group (Fig. 5A,B) whereas it does not affect the binding of haloxyfop with its smaller monocyclic 3-chloro-5-(trifluoromethyl)pyridin-2-yl group (Fig. 5C,D). This was further supported by computational binding energy calculation. The mutation increased the calculated binding energy of quizalofop, relative to the wildtype, whereas this same mutation lowered the calculated binding energy of haloxyfop. Follow-up experiments confirmed these computational observations, where quizalofop-resistant genotypes are more sensitive to haloxyfop than the wildtype genotype in both whole-plant (Table 1) and enzyme-level (Table 2) dose–response studies. The leftward shift of whole-plant (Fig. 1B) and enzyme activity (Fig. 3B) dose–response curves with an increasing number of resistance mutations indicates that the quizalofop resistance mutation imparts negative cross-resistance to haloxyfop. The negative cross-resistance to haloxyfop is not concerning for the CoAXium® Wheat Production System because the system is only available in the United States, where haloxyfop is not labeled for use in wheat.

In conclusion, the CoAXium wheat production system is a new tool for management of difficult to control winter weeds^7,18^. The AXigen® resistance trait is based on an alanine to valine mutation at position 2004 of ACCase which hinders the binding of quizalofop. An unexpected outcome of this research is the discovery that the AXigen trait imparts negative cross-resistance to haloxyfop. Further studies could identify other group 1 inhibitors that may be developed as tools to efficiently control volunteer CoAXium wheat.

## Methods

### Plant material and growing conditions

Four wheat genotypes were used to compare whole-plant resistance of lines with wildtype and mutant ACCase as well as specific activity and resistance of extracted ACCase. The AF10 and Hatcher lines were provided by Dr. Scott Haley from the Department of Crop and Soil Science, and the varieties Byrd, and Incline AX were obtained from the Colorado Wheat Research Foundation. All experiments carried out on these wheat varieties were done with their permission and complied with local and national regulations. Byrd is a quizalofop-susceptible variety that was the recurrent parent for Incline AX, a CoAXium® variety, whereas Hatcher is the original mutagenized variety. AF10 is one of the initial quizalofop-resistant lines, which contains a homozygous mutant *ACC1* homoeolog in sub-genome D^10^. Incline AX exhibits two mutant homoeologs in sub-genomes A and D. Byrd was the wildtype, null mutant in all physical experiments except for the quizalofop plant growth dose–response experiment, where Hatcher was substituted. AF10 and Incline AX were consistently used as representative lines with the quizalofop-resistance trait in one and two genomes, respectively, in the same experiments. Plants for the whole-plant dose responses and ACCase extractions were grown from seed in soilless media. Plants were maintained in a greenhouse environment with temperatures from 20 to 25 °C, relative humidity between 50 and 70%, and a 14-h daylength with natural lighting supplemented by sodium halide lamps.

### Herbicide dose effect on plant growth

Prior to reaching the jointing stage, wheat seedlings were treated with herbicide. Untreated seedlings of each line were also retained to normalize data. Eleven rates of herbicide were used in each dose–response experiment. Quizalofop herbicide doses for the susceptible line ranged from 3.85 to 108 g ae ha^−1^, whereas doses for resistant lines ranged from 15.4 to 493 g ae ha^−1^. Haloxyfop doses ranged from 15.4 to 616 g ae ha^−1^ for all wheat lines. Non-ionic surfactant was added to each dose (0.25% v/v). All treatments were applied in a single pass at 40 cm above the plant canopy using a spray chamber. Each treatment was triplicated, with four plants comprising a replicate. Fresh leaf biomass was harvested three weeks after treatment. Biomass was adjusted in some instances to account for germination of less than four plants per replicate.

### Enzyme extraction

Approximately 5.0 g of crown tissue was harvested from each genotype at the two-leaf stage, flash frozen in liquid nitrogen, and stored at − 80 °C. Enzyme extraction and activity assay were modified from previously published methods^19–21^. Frozen wheat tissue was powdered in liquid nitrogen. All subsequent extraction steps were carried out on ice or in a cold room to maintain an extract temperature of 4 °C. Ground tissue was transferred to 14.85 mL extraction buffer (100 mM Trizma, 20 mM DTT, 2 mM l-ascorbic acid, 1 mM EDTA, 0.5% w/v PVP-40, 0.5% w/v insoluble PVP-20, and 10% v/v glycerol; pH 8.0) and 150 µL of 100 mM PMSF (dissolved in 100% ethanol). The tissue was homogenized at max speed for 30 s with a homogenizer probe. Homogenate was filtered by hand with Miracloth, followed by centrifugation for 30 min at a speed of 25,000× g. The resulting supernatant was slowly brought to 66% saturation with solid ammonium sulfate and stirred for an hour.

Ammonium sulfate solutions were centrifuged using the same parameters as the previous centrifugation step to precipitate ACCase and similar proteins. After discarding the supernatant, the protein pellets were resuspended with 2.5 mL elution buffer (50 mM tricine, 50 mM KCl, 2.5 mM MgCl_2_, and 1 mM DTT; pH = 8.0). The protein extracts were desalted using a gravity protocol with conditioned PD-10 columns. Desalted protein extracts were eluted with 3.5 mL of elution buffer and mixed with 25% v/v glycerol.

### Enzyme specific activity assay

To determine ACCase specific activity, four 40 µL aliquots of fresh protein extract per genotype were preincubated for 3 min in assay solution without acetyl-CoA. Acetyl-CoA was subsequently added to three aliquots to initiate enzyme activity, whereas water was added in lieu of acetyl-CoA for a background readings. Preincubation and reaction steps were maintained at 32 °C with moderate shaking. Final concentrations of assay solution components for each 200 µL reaction (including enzyme aliquot) were as follows: 20 mM tricine (pH = 8.3), 10 mM KCl, 10 mM MgCl_2_, 5 mM ATP, 3.24 mM NaHCO_3_, 2.5 mM DTT, 0.1% w/v BSA, 0.25 mM acetyl-CoA, and 0.25 mM NaH^14^CO_3_ (provides 18.5 kBq per reaction). After 10 min, enzyme reactions were quenched with 20 µL of 12 M hydrochloric acid. Solutions were transferred to 20 mL scintillation vials and left uncapped overnight to enable volatilization of unincorporated carbonate to CO_2_. Reactions were carried out in a fume hood equipped with a ^14^C filter. The following day, 10 mL of scintillation cocktail was added to each vial. Vials were capped and vortexed for 20 s prior to analysis. Radioactivity was measured with a liquid scintillation analyzer in disintegrations per min (DPM).

### Herbicide dose effect on ACCase activity

Specific activity dose-responses were conducted using the same protocol as the specific activity assay but with the addition of herbicide immediately preceding the preincubation step. Dilution series were prepared with quizalofop acid and haloxyfop acid analytical standards dissolved in 100% acetonitrile. Triplicated dose treatments per genotype included background readings (water added in lieu of acetyl-CoA), null herbicide doses, and doses that provided 0.0100, 0.100, 1.00, 10.0, 100, and 1000 µM herbicide in the final assay solution.

### Homology modeling of wheat carboxyl transferase domain

The segments of wheat ACCase sequence where the A2004V substitution is located were aligned to the sequence of the CT domain of yeast ACCase using EMBOSS Needle^22^. This information was used to build a homology model of wheat ACCase based on the crystal coordinates of the carboxyl transferase (CT) domain of yeast ACCase (1UYS)^12^ available from the RCSB protein data bank. The homology modeling pipeline was similar to that reported previously to model the binding of glufosinate on resistant glutamine synthetase from *Lolium perenne*^23^. A preliminary model was obtained using Modeller 10.0^24–26^. The model was assembled in its functional dimer configuration and refined using GROMACS (version 2018.3)^27,28^ on a workstation with two processors (96 threads) and video cards (10 GB GPU memory each). Steric clashes or inappropriate geometries were corrected through molecular dynamics simulation and evaluated using MolProbity^29,30^ as described before.

Proteins and ligand interactions were visualized using PyMOL v.2.3.3^31^. Additionally, the binding of quizalofop and haloxyfop to native and mutant carboxyl transferase dimers of wheat was measured using established protocols developed for Autodock^32,33^. Briefly, the receptor was defined to encompass residues lining the herbicide binding domain. Amino acid charges were calculated using Gasteiger charges including all polar hydrogens^34–36^. The docking procedure imposed a covalent map to involve the nitrogen hydrogen of ILE 222 in the docking of the herbicides within the gridbox. A total of 100 poses were generated.

### Statistical analysis

All statistical analysis was conducted with packages in RStudio v.1.2.5033 with R "Orange Blossom”^28^. To model the effect of herbicide on plant growth, fresh weight data was first normalized to percent of control means. Data was transformed with a Box-Cox transformation. For quizalofop data, a two-parameter log-logistic regression was fit using the LL.4 [drc] function:

1 $$y = c + \frac{d - c}{{1 + {\text{exp}}\left( {m\left[ {\log \left( x \right) - \tilde{e}} \right]} \right)}}$$

where lower limit *c* is fixed at 0, upper limit *d* is fixed at 100, *m* is the slope, and *e* is the 50% growth reducing dose (GR_50_). A four-parameter Brain-Cousens model was fit using the BC.4 [drc] function for haloxyfop data:

2 $$y = c + \frac{d - c + f}{{1 + {\text{exp}}\left( {m\left[ {\log \left( x \right) - \tilde{e}} \right]} \right)}}$$

where lower limit *c* is fixed at 0, *d* is the upper limit*, f* is the hormesis effect, with *m* and *e* as additional parameters without direct interpretation. The GR_50_ of the haloxyfop model was estimated through parameterization with the ED [drc] function. A resistance factor (R:S_50_) was calculated by dividing GR_50_’s of quizalofop-resistant wheat lines by the GR_50_ of a wildtype, susceptible line with the EDcomp [drc] function. Student’s t-tests (a = 0.05) of resistance factor ratios were used to identify significant fold differences in resistance between estimated GR_50_ doses.

A linear calibration curve of absorbance versus BSA concentration was fit using the stats [lm] function to estimate wheat ACCase extract protein concentrations^36^:

3 $$y = mx + b$$

where *m* and *b* constants and standard deviations were 2.76·10^–3^ ± 3.21·10^–4^ and 1.40·10^–1^ ± 1.32·10^–3^, respectively. The residual standard error of the calibration was 1.06·10^–3^ with a multiple R-squared value of 0.987. Genotype protein concentrations were calculated from the calibration and adjusted for dilution.

Per genotype, the DPM measurement of a reaction to which water was added instead of acetyl-CoA was subtracted from replicate specific activity measurements as background activity. Mean DPM enzyme specific activity measurements were converted to ^14^C pmol units using manufacturer provided activity of ^14^C-labeled sodium bicarbonate. Specific activity was then weighted by protein concentration to yield units of ^14^C pmol mg^−1^ protein. Standard errors reflect error propagation of DPM measurement replicates and protein concentration. Specific activity was compared between genotypes using ordinary one-way ANOVA F-protected Student’s t-tests (a = 0.05, n = 3) with anova [stats] and emmeans [emmeans] functions.

For specific activity dose–response analysis, background activity was subtracted from DPM readings. Background corrected DPM readings were normalized to percent residual activity using control means for each genotype. Data was modelled using the same methods as for the effect of quizalofop on whole-plant growth, with the exception that *e* represents the 50% enzyme inhibition dose (I_50_). Similar resistance factors (R:S) were calculated using I_50_ estimates, followed by Student’s t-tests (a = 0.05) of ratios to identify significant fold differences in resistance between estimated doses.

## Data Availability

The datasets generated during and/or analyzed during the current study are available from the corresponding author on reasonable request.
